# Efficacy of the interpersonal and social rhythm therapy (IPSRT) in patients with bipolar disorder: results from a real-world, controlled trial

**DOI:** 10.1186/s12991-020-00266-7

**Published:** 2020-03-09

**Authors:** Luca Steardo, Mario Luciano, Gaia Sampogna, Francesca Zinno, Pasquale Saviano, Filippo Staltari, Cristina Segura Garcia, Pasquale De Fazio, Andrea Fiorillo

**Affiliations:** 1 Department of Psychiatry, University of Campania “Luigi Vanvitelli”, Largo Madonna Delle Grazie, 80138 Naples, Italy; 2 Psychiatric Unit, Department of Health Sciences, University Magna Graecia, Catanzaro, Italy; 3 UOSM Nola, DSM ASL NA 3 Sud, Nola, Italy; 4 Department of Medical and Surgical Sciences, University Magna Graecia of Catanzaro, Catanzaro, Italy

**Keywords:** Bipolar disorder, Psychosocial intervention, Affective morbidity index, Treatment strategy

## Abstract

**Background:**

Bipolar disorder (BD) is one of the most burdensome mental disorders, with a lifetime prevalence of 2.4%, with a prevalence of 0.6% for bipolar type I and 0.4% for bipolar type II. Several interventions have been developed to implement the treatment strategy of bipolar disorder, including the Interpersonal and Social Rhythm Therapy (IPSRT). This intervention has been specifically developed to manage patients’ stressful life events, improve the disruptions of social and circadian rhythms and increase adherence to medications. The aim of the present study is to assess the efficacy of IPSRT on affective and anxiety psychopathology, social functioning, response to pharmacological treatment and affective morbidity index (AMI) in BD patients.

**Methods:**

BD patients were consecutively recruited at the Mood Disorder Unit of the University of Campania “Luigi Vanvitelli” and randomly assigned to the experimental group receiving the IPSRT or to the Treatment as Usual (TAU) group. Patients were assessed at baseline, after 3 and 6 months with several validated assessment tools and with the affective morbidity index.

**Results:**

At the end of the intervention, compared to controls, patients from the experimental group reported a significant improvement in anxious depressive and manic symptomatology, global functioning; and response to mood stabilizers. Patients in the IPSRT group reported a reduction at the AMI score.

**Conclusions:**

IPSRT has been confirmed to be effective in improving the clinical symptomology of BD patients and in improving the affective morbidity index. Further studies with longer follow-up are needed in order to assess the stability of the results.

*Trial registration* The study was approved by the local ethical review board (N001567/28.01.2018)

## Introduction

Bipolar disorder is one of the most burdensome mental disorders, with a lifetime prevalence of 2.4%, and a prevalence of 0.6% for bipolar I and 0.4% for bipolar II subtypes [1]. It poses a significant burden on patients, their relatives and the society at large. It is characterized by mood swings in both polarities, manic and depressive, and heterogeneous symptoms, involving affective, cognitive and physical alterations. Its management includes pharmacological agents such as mood stabilizers, second-generation antipsychotics, antidepressants as well as psychosocial interventions, including cognitive behavioral therapy (CBT) [2, 3], psychoeducation (PE) [4–8], family-focused therapy (FFT) and interpersonal and social rhythm therapy (IPSRT) [9–11]. Although pharmacotherapy is the recommended first-line therapy for manic, depressive and residual states, medication adherence is typically poor, relapse rates are high, and full remission is not always achieved. Psychosocial therapies have been found to be effective in terms of improvement of medication adherence, identification of early warning signs, increasing in self-management skills and in family communication, all elements which could potentially explain the reduced rate of relapses in patients treated with these interventions. Therefore, the integration between pharmacological and psychosocial interventions is essential for the optimal management of patients with bipolar disorders and should be implemented in the clinical routine care [12]. In fact, several international guidelines [13–16] recommend their use as adjunctive interventions to pharmacological treatment for the long-term management of bipolar disorder in both the acute and maintenance phases [17, 18]. These different approaches share several aims, including: (1) the improvement of sleep disturbances; (2) the promotion of healthy lifestyle behaviors; (3) the monitoring of mood shifts; (4) the early recognition of patients’ warning signs; (5) the improvement of problem-oriented coping strategies [19].

IPSRT has been specifically developed to manage patients’ stressful life events, improve the disruptions of social and circadian rhythms and increase their adherence to medications [20]. The IPSRT is based on the theoretical approaches of the interpersonal psychotherapy (IPT) and social rhythm therapy. The efficacy of IPSRT on the outcome of BD patients has been tested only in a handful of studies [21–25]. Frank et al. [20] found that IPSRT is associated with a better outcome in terms of remission from manic symptoms in a cohort of 44 BD patients, compared to patients receiving pharmacotherapy only. Furthermore, in a RCT carried out by the same authors [26], the IPSRT was associated with a longer euthymic period compared to patients from the control group.

In this study, we evaluated the efficacy of IPSRT in terms of reduction of levels of patients’ affective and anxiety psychopathology, social functioning, response to pharmacological treatment and affective morbidity index (AMI), compared to a group of patients receiving standard care.

## Methods

### Participants

The study has been conducted at the Mood Disorder Unit of the University of Campania “Luigi Vanvitelli” in Naples (Italy) during the period January 2018 to February 2019.

Patients were included in the study if they met the following criteria: (1) age between 18 and 70 years; (2) a DSM-5 diagnosis of type-I or type-II bipolar disorder, confirmed through Structured Clinical Interview for DSM-5 disorders, clinician version—SCID-5-CV [27]; (3) stable treatment with mood stabilizers (at least 1 year duration and, in the case of lithium or valproic acid, at therapeutic blood levels); (4) willingness to participate in the study, expressed by written informed consent provided upon complete description of the protocol. Patients were excluded in case of: (1) inability to give a written consent to participate in the study; (2) diagnosis of any neurologic disease; (3) presence of drug and/or alcohol abuse; (4) receiving any other psychotherapeutic intervention at the moment of the recruitment.

Patients were consecutively recruited and randomly assigned to the experimental group receiving the IPSRT or to the control group, receiving the pharmacotherapy treatment plus treatment as usual.

The study has been carried out in accordance with the latest version of the Declaration of Helsinki. All patients gave their written informed consent and were enrolled in the study.

The study was approved by the local ethical review board (N001567/28.01.2018).

### Procedures

Patients’ socio-demographic (i.e., gender, age at study entry, employment status and level of education) and clinical characteristics (i.e., age at onset, age at first psychiatric contact, duration of illness, lifetime number of affective episodes, pattern of illness course, presence of mixed affective states, and number of suicidal attempts) were recorded with an ad hoc schedule. Clinical course and treatment history for each patient were evaluated through the National Institute of Mental Health Life Chart Method at baseline [18]. Patients have been assessed at baseline (T0), and after 3 (T1) and 6 months (T2).

### Psychopathological assessments

All recruited patients were assessed through the administration of: (a) the Inventory of Depressive Symptomatology (Self-Report) (IDS-SR) [28]; (b) the Montgomery Asberg Depression Rating scale (MADRS) [29]; (c) the 14-item Hamilton Rating Scale for Anxiety (HAM-A) [30]; (d) the Mania Rating Scale (MRS) [31]; (e) the global assessment of functioning (GAF) [32]. Two psychometric tools have been adopted for assessing depressive symptoms, in order to obtain an objective assessment of depressive symptomatology as well as the personal perception of patients, through a self-report scale.

Patients’ response to mood stabilizers was assessed by the Retrospective Criteria of Long-term Treatment Response in Bipolar Disorder (the ALDA Scale), which consists of two criteria: (a) association between clinical improvement and treatment; and (b) degree of the causal relationship between clinical improvement and prophylactic treatment. A total score was obtained by subtracting the (b) from the (a) score [33, 34].

The clinical course of BD was assessed with the Affective Morbidity Index (AMI; [35, 36]), which considers the duration and the severity of mood episodes. Symptoms’ severity is rated as follows: (1) symptoms not requiring any treatment modification; (2) symptoms requiring an adjunctive pharmacological intervention, but not a dosage change of mood stabilizers; (3) symptoms requiring treatment modification to be managed within hospitalization. AMI absolute value was calculated according to the following formula: AMI = (weeks with degree 1) + (weeks with degree 2) × 2 + (weeks with degree 3) × 3/total number of weeks × 3.

The AMI was calculated for the first 3 months (AMI-T1), from the third until the sixth month (AMI-T2) and during the whole treatment period (AMI_tot_). The variation of the AMI index was considered a measure of psychological improvement.

### The interpersonal and social rhythm therapy

IPSRT is a psychosocial intervention specifically developed to address circadian dysregulation in BD and it is based on the principles of social rhythm therapy, interpersonal psychotherapy and psychoeducation [20]. The intervention focuses on the four main areas of interpersonal psychotherapy (grief, role of transition, role of disputes and interpersonal deficits) combined with strategies to improve the social and circadian rhythms. In addition, psychoeducational sessions are provided to improve patients’ compliance to pharmacological treatments. It consists of 12 weekly sessions, each lasting about 90 min. All participating patients were asked to fill in the social rhythm metric scale.

The intervention is divided in four phases: (1) the initial two sessions are focused on illness history and aim to identify the relationship between stressful life events and mood shifts; (2) the second phase includes four sessions focused on the reorganization of social rhythms and the increase of skills to cope with social stressors; (3) the maintenance phase (four sessions) focuses on reinforcing new social rhythms and building confidence in learned techniques in order to prevent future affective episodes; (4) the final phase consists of two sessions, during which the skills achieved with the IPSRT are further discussed and advices for the future are given.

All patients received individual sessions of IPSRT. The intervention was provided by a trained psychiatrist (LS).

### Statistical analyses

Descriptive statistics included frequencies and means, as appropriate. Differences between the two groups were explored through Chi-squared and *t*-tests, as appropriate. The General Linear Model (GLM)-Repeated Measures test was used to explore variations of HAM-A, MRS, IDSSR, MADRS and GAF in both groups in the 6-month period (*T*_0_, *T*_1_ and *T*_2_). Eta-squared, a measure of effect size in ANOVA, was calculated for significant results. The AMI was calculated as the sum of the weeks of degree 1, weeks with degree two multiplied for two, weeks with degree three multiplied for three divided for the total number of weeks multiplied for three. The level of statistical significance was set at *p* < 0.05. Data analyses have been made with the Statistical Package for Social Sciences Version 21 (SPSS, Chicago, Illinois, USA).

## Results

The final sample consists of 44 BD patients, who were randomly allocated to the experimental or to the control group. All patients allocated in the IPSRT group attended all sessions of the intervention, with 100% retention rate.

The main socio-demographic and clinical characteristics of recruited patients are reported in Table 1. The two samples did not differ with regard to socio-demographic and clinical variables, with the only exception of diagnosis distribution and clinical course. In the experimental group, 8 patients were treated with valproate (900 mg/day), one with lithium (900 mg/day), 8 with lithium in combination with antipsychotics and 5 with valproate in combination with other medications. In the control group, 5 patients were treated with valproate (900 mg/day), 10 with lithium (900 mg/day), 7 with lithium in combination with antipsychotics. Differences between the two groups emerged about the diagnosis (*χ*^2^ = 5,350; *df * = 1 *p * = 0.021) and clinical course (*χ*^2^ = 14,186; *df * = 3; *p* = 0.003); no statistical differences have been found for all other considered variables.

**Table 1:** Socio-demographic and clinical characteristics of the global sample

	IPSRT group		TAU group		Group comparison	*df*	*p*
Sex (male, %)	10	46	9	41	0.093	1	0.761
Age (M ± SD)^a^	49.1	12.6	46.7	12.8	*t* = 0.629	42	0.533
Civil status^b^							
Single	14	64	10	46	*χ*^2^ = 2.889	2	0.236
Married	8	36	10	46			
Divorced	0	0	2	9			
Education (years)^a^	13.9	3.7	13.6	3,2	*t *= 0.263	42	0.794
Employed (yes)^b^	11	50	14	64.6	*χ*^2^ = 0.834	1	0.361
Diagnosis^b^							
BD I	19	86	12	55	*χ*^2^ = 5.350	1	0.021
BD II	2	14	10	46			
Family psychiatric history (yes)^b^	15	68	14	64	*χ*^2^ = 0.101	1	0.750
Family history for other disorders (yes)^b^	7	32	11	50	*χ*^2^ = 1.504	1	0.220
Comorbidity (yes)^b^	8	36	12	55	*χ*^2^ = 1.467	1	0.226
Age at onset^a^	28.5	8.8	28.8	14.8	*t* = − 0.099	42	0.921
Age at first psychiatric visit^a^	28.5	8.7	32	13.9	*t* = − 0.987	42	0.329
Age at first hospitalization^a^	33.1	13.2	28	8.8	*t *= 0.944	17	0.358
Age at first depressive episode^a^	29	9.8	28.8	13.5	*t *= 0.056	40	0.955
Age at first manic episode^a^	32	13.3	28.1	7.9	*t *= 0.921	29	0.365
Age first hypomanic episode^a^	31.1	9.4	34.1	14.3	*t *= − 0.623	25	0.539
Course^b^							
MDI	12	55	11	50	*χ*^2^ = 14.186	3	0.003
MID	3	14	4	18			
DMI	7	32	0	0			
IRR	0	0	7	32			
Number of depressive episodes^a^	4.1	3.6	3.7	3.5	*t *= 0.345	40	0.732
Number of manic episodes^a^	2.5	2.1	2.9	2.6	*t* = − 0.552	32	0.585
Number of hypomanic episodes^a^	1,7	2,3	2.8	2.2	*t *= − 1.564	36	0.127
Suicide attempts (yes)^b^	2	9	2	9	*χ*^2^ = 0	1	1.000
Psychotic symptoms (yes)^b^	12	55	9	41	*χ*^2^ = 0.820	1	0.365
Aggressiveness (yes)^b^	15	68	13	59	*χ*^2^ = 0.393	1	0.531
Number depressive episode last year^b^							
0	5	23	6	27	*χ*^2^ = 1.091	2	0.580
1	15	68	12	55			
2	2	9	4	18			

*BD I *bipolar disorder type I, *BD II* bipolar disorder type II, *MDI* manic–depression interval, *MID* manic–interval depression, *DMI* depression–manic interval, *IRR* irregular

^a^ Data are expressed as mean and standard deviations

^b^ Data are expressed as frequencies and percentages

**Fig. 1:**
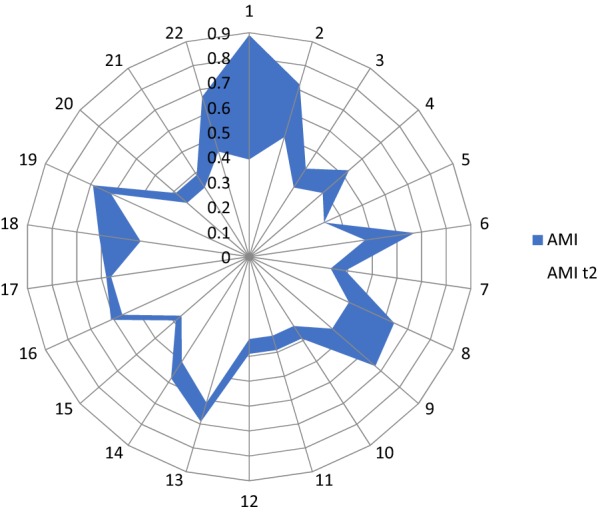
Reduction of AMI index in patients’ experimental group

Data on the efficacy of the IPSRT on AMI index are reported in Table 2 and in Fig. 1; the results of GLM repeated measures are reported in Table 2. At the end of the intervention, compared to controls, patients from the experimental group reported a significant improvement in anxious symptoms (HAM-A: *p* < 0.001;* ƞ*^2^ = 0.389), manic symptoms (MRS: *p* < 0.004; * ƞ*^2^ = 0.234), depressive symptoms (IDSSR: *p *  < 0.007; * ƞ*^2^ = 0.216; MADRS: *p* < 0.057), global functioning (GAF: *p* < 0.001; * ƞ*^2^ = 0.309); and response to mood stabilizers (ALDA scale: * p*< 0.001; * ƞ*^2^ = 0.300). Moreover, patients receiving the experimental intervention also had an improvement in psychological functioning, as confirmed by the reduction in the AMI score (*p* < 0.001; * ƞ*^2^ = 0.341).

**Table 2:** Differences at total scores’ scales between the two groups at baseline, after 3 and 6 months

	Group	*T*_0_	*T*_1_	*T*_2_	*F*	*p*	*ƞ*^2a^
HAM-A	Group 1	8.636	1.773	0.545	Time: *F*(2, 41) = 0.816	.449	
	Group 2	5.773	13.455	17.773	Group:* F*(2, 41) = 13.076	< .001	0.389
MRS	Group 1	9.864	0.636	0.727	Time: *F*(2, 41) = 2.428	.101	
	Group 2	5.500	7.273	8.091	Group: *F*(2, 41) = 6.275	.004	0.234
IDSSR	Group 1	16.273	11.318	3.727	Time: *F*(2, 41) = 1.099	.343	
	Group 2	15.136	28.955	25.273	Group: *F*(2, 41) = 5.655	.007	0.216
MADRS	Group 1	7.636	4.182	1.545	Time: *F*(2, 41) = 0.796	.458	
	Group 2	6.364	8.955	8.318	Group: *F*(2, 41) = 3.073	.057	
GAF	Group 1	67.636	78.091	78.500	Time: *F*(2, 41) = 1.293	.285	
	Group 2	73.864	68.909	69.227	Group: *F*(2, 41) = 9.170	.001	0.309
ALDA scale	Group 1	4.818	5.091	5.273	Time: *F*(2, 41) = 1.187	.315	
	Group 2	4.864	4.273	4.227	Group: *F*(2, 41) = 8.792	.001	0.300
AMI	Group 1		0.547	0.437	Time: *F*(2, 41) = 21.700	< .001	0.341
	Group 2		0.713	0.713	Group: *F*(2, 41) = 21.700	< .001	0.341

*Group 1* PSRT group, *Group 2* control group

## Discussion

One of the main findings of our study is the improvement of manic, anxiety and depressive symptoms in patients receiving the IPSRT compared to controls [37]. The reduction of psychopathological burden remained stable after 6 months in the experimental group. The intervention was well-received by patients and was associated with a high retention rate, as highlighted by the fact that none of the recruited patients dropped out [38, 39]. Moreover, the IPSRT can be easily implemented in the routine care of mental health centers considering that it can be administered by all categories of mental health professionals (including psychiatrists, psychologists, nurses, social workers and psychiatric rehabilitation technicians) after a brief training [40, 41]. The positive effect of the intervention on all symptom domains can be explained by several factors, including that the strong relationship between the improvement of circadian rhythms and symptomatic remission in patients with BDs [42–44]. In fact, sleep dysregulation is considered a trigger factor for the development of manic or depressive symptoms [45, 46]. The IPSRT has a specific focus on sleep disturbances through the monitoring of daily levels of energy, thus contributing to a better daily planning. Another possible explanation for the improvement of the levels of psychiatric symptoms is the fact that our intervention included sessions on the improvement of patients’ compliance to psychopharmacological medications [47, 48]. In particular, information on possible side effects of medications are regularly provided to patients and their concerns about medications and side effects are regularly addressed, during the treatment period.

Another relevant finding of our study is the improvement of anxiety symptoms. Anxiety is very common in BD patient and is associated with poor treatment response [49]. The IPSRT contributes to reduce the levels of anxiety by helping patients to address their interpersonal deficits and improving their emotional dysregulation, and not just by managing affective symptoms. Similar results have been reported with other psychotherapeutic approaches, such as cognitive behavioral therapy (CBT) and mindfulness-based cognitive therapy (MCBT) [50]. As expected, at the follow-up we observed an improvement of GAF score. This result emphasizes the importance of the interpersonal intervention in improving all aspects of patients’ life, thus contributing to prevent mood shifts [51].

The differences between the two groups at the Alda scale total score can be due to the improvement of patients’ adherence to medications following their participation at the IPSRT.

In our study, the provision of IPSRT significantly improved the AMI index. To the best of our knowledge, this is the first study which tested the efficacy of a psychosocial intervention in BD through AMI. This instrument gives an accurate picture of psychopathological status as it assesses the severity and length of mood shifts over the lifetime [52].

Our results confirm the importance of complementing the pharmacological treatment with psychotherapy in bipolar disorder in a naturalistic setting [7, 8]. Our patients treated with IPSRT plus pharmacotherapy reported an improvement of affective and anxiety symptoms without requiring any dose adjustment of mood stabilizers. This result, confirmed by the reduction of AMI index at 3 and 6 months, emphasizes the importance of IPSRT in reducing the psychological burden of bipolar disorder and preventing mood relapses [53, 54].

This study has some limitations that need to be acknowledged. In particular, the sample size was small and the follow-up was short. In fact, to establish a strong association between IPSRT and mood improvement, longer follow-ups (i.e., at 12 and 18 months) with larger samples are needed. Moreover, the adoption of the MRS for evaluating manic symptoms should limit the generalizability and external validity of our findings. However, this is a not funded study conducted in the clinical routine care and we decide to use an assessment instrument routinely used in the bipolar unit of the mental health department, in order to reduce the burden for participating professionals.

Furthermore, patients’ satisfaction with the received treatment has not been measured through ad hoc validated instrument. Our study can be considered as a pilot and we are planning a follow-up study with the same cohort of patients in order to verify if our encouraging results at 6 months are confirmed and for evaluating patients’ expectations and levels of satisfaction with the received treatment.

## Conclusions

The main finding of the present study is the reduction of the psychopathological burden in BD patients treated with IPSRT, as shown by the reduction of total score scale after 6 months and changing in affective morbidity index. Moreover, this intervention has been well received by patients and it is feasible in clinical naturalistic setting. Further studies should be conducted in order to evaluate in long-term efficacy in terms on reduction of relapses.

## Data Availability

All data generated or analyzed during this study are included in this published article.
