# Correction to: Efficacy of the interpersonal and social rhythm therapy (IPSRT) in patients with bipolar disorder: results from a real-world, controlled trial

**DOI:** 10.1186/s12991-020-00278-3

**Published:** 2020-04-18

**Authors:** Luca Steardo, Mario Luciano, Gaia Sampogna, Francesca Zinno, Pasquale Saviano, Filippo Staltari, Cristina Segura Garcia, Pasquale De Fazio, Andrea Fiorillo

**Affiliations:** 1 Department of Psychiatry, University of Campania “Luigi Vanvitelli”, Largo Madonna Delle Grazie, 80138 Naples, Italy; 2 Psychiatric Unit, Department of Health Sciences, University Magna Graecia, Catanzaro, Italy; 3 UOSM Nola, DSM ASL NA 3 Sud, Nola, Italy; 4 Department of Medical and Surgical Sciences, University Magna Graecia of Catanzaro, Catanzaro, Italy

## Correction to: Ann Gen Psychiatry (2020) 19:15 10.1186/s12991-020-00266-7

Following publication of the original article [1], the authors reported an error in the legend of Table 2.

It should be “Group 1: control group, Group 2: IPSRT group” instead of “Group 1 PSRT, Group 2 control group”.

The original article [1] has been updated.
